# Fatty Acids and Stomach Contents Reveal Dietary Overlaps of Two Prospering Squid Species in the North Sea

**DOI:** 10.1002/ece3.72464

**Published:** 2025-11-28

**Authors:** Hanna Rittinghaus, Reinhard Saborowski, Ulrike K. R. Kammann, Daniel Oesterwind, Anne F. Sell

**Affiliations:** ^1^ Thünen Institute of Sea Fisheries Bremerhaven Germany; ^2^ Faculty 2: Biology/Chemistry University of Bremen Bremen Germany; ^3^ Alfred Wegener Institute, Helmholtz Centre for Polar and Marine Research Bremerhaven Germany; ^4^ Thünen Institute of Fisheries Ecology Bremerhaven Germany; ^5^ Thünen Institute of Baltic Sea Fisheries Rostock Germany

**Keywords:** Cephalopoda, fatty acid trophic markers, feeding ecology, *Illex coindetii*, *Loligo forbesii*

## Abstract

Worldwide, the distribution ranges of cephalopods are expanding due to climate change. In the North Sea, the broadtail shortfin squid *Illex coindetii* (Ommastrephidae) has recently established a successful breeding population and now coexists with the local veined squid *Loligo forbesii* (Loliginidae). Among other squids, both species are potential food competitors. To investigate the trophic preferences of either species, we analyzed the fatty acid (FA) composition of the mantle tissue and the digestive gland (DG) via gas chromatography (GC). According to the FA trophic marker concept, FA signatures may be indicative of the staple food over a longer period. Additionally, we investigated the stomach contents of both species visually to obtain information about their recently consumed food. The FA patterns of the DG of both species suggested moderate overlaps of the prey spectra. The stomach contents of *I. coindetii* and *L. forbesii* showed significant overlaps, further confirmed by the trophic index Pianka's niche overlap. Both squid species fed on commercially important taxa, including herring, cod, and decapods. The population of *I. coindetii* in the North Sea will rise along with warming. As a consequence, competition with *L. forbesii* for food will increase, and food web interactions will change. Predation between both squid species may increase as well.

## Graphical abstract

Distribution ranges of cephalopods are expanding worldwide and their abundances are increasing due to ocean warming. Recently, the broadtail shortfin squid *Illex coindetii* (Ommastrephidae) expanded into the North Sea where it established a successful spawning stock beside the resident veined squid *Loligo forbesii* (Loliginidae). Stomach content analysis and FA analysis of the DG showed significant similarities and overlaps in the prey spectra of both species and confirmed food competition.

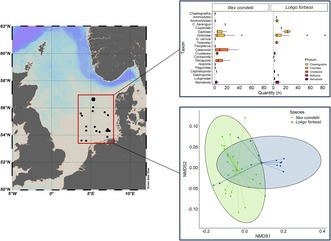

## 1 Introduction

Cephalopods are ubiquitous and highly adaptable predators that inhabit virtually all depth strata from coastal surface waters to the deep sea. Among the cephalopods, the squid—consisting of the orders Myopsida and Oegopsida—are the most diverse and commercially and ecologically most important group (WoRMS Editorial Board 2025, Barrett et al. 2022). Worldwide, the distribution ranges of cephalopods are expanding and their abundances are increasing (Barrett et al. 2022). Ocean warming caused by anthropogenic climate change seems to be one of the major drivers of this trend (Doubleday et al. 2016; van der Kooij et al. 2016; Oesterwind et al. 2022). Additionally, overfishing of finfish stocks also seems to have positively affected cephalopod populations by diminishing both potential predators and food competitors (Piatkowski et al. 2001; Doubleday et al. 2016). Thus, where fish stocks have been overexploited, cephalopods may have the potential to fill the vacant trophic niches and may locally even have the potential to replace fishes as a commercial fisheries resource (Rodhouse and Nigmatullin 1996).

The North Sea is a shallow shelf sea in the North‐Eastern Atlantic. The water temperature in this region is increasing at a much higher rate than the global average (Núñez‐Riboni and Akimova 2015). This warming has led to a regime shift from cold‐temperate to warm temperate in the late 1980s and a shift toward more oceanic conditions, causing changes in abundances and distribution ranges in many taxa across the food web (Reid et al. 2003; van der Kooij et al. 2016; Saborowski and Hünerlage 2022). As a result, the conditions in the North Sea have become less favorable for cold‐water demersal fish species, which shifted their distribution areas northwards or to deeper waters (Reid et al. 2003; Dulvy et al. 2008; van der Kooij et al. 2016). The new conditions allowed expansion or immigration of warm‐temperate species. Among the winners of this regime shift are squids, whose stocks and distribution ranges have since increased (van der Kooij et al. 2016; Oesterwind et al. 2020). However, the feeding ecology of North Sea squid is not well understood (Oesterwind et al. 2022; Oesterwind and Piatkowski 2023).

**FIGURE 1:**
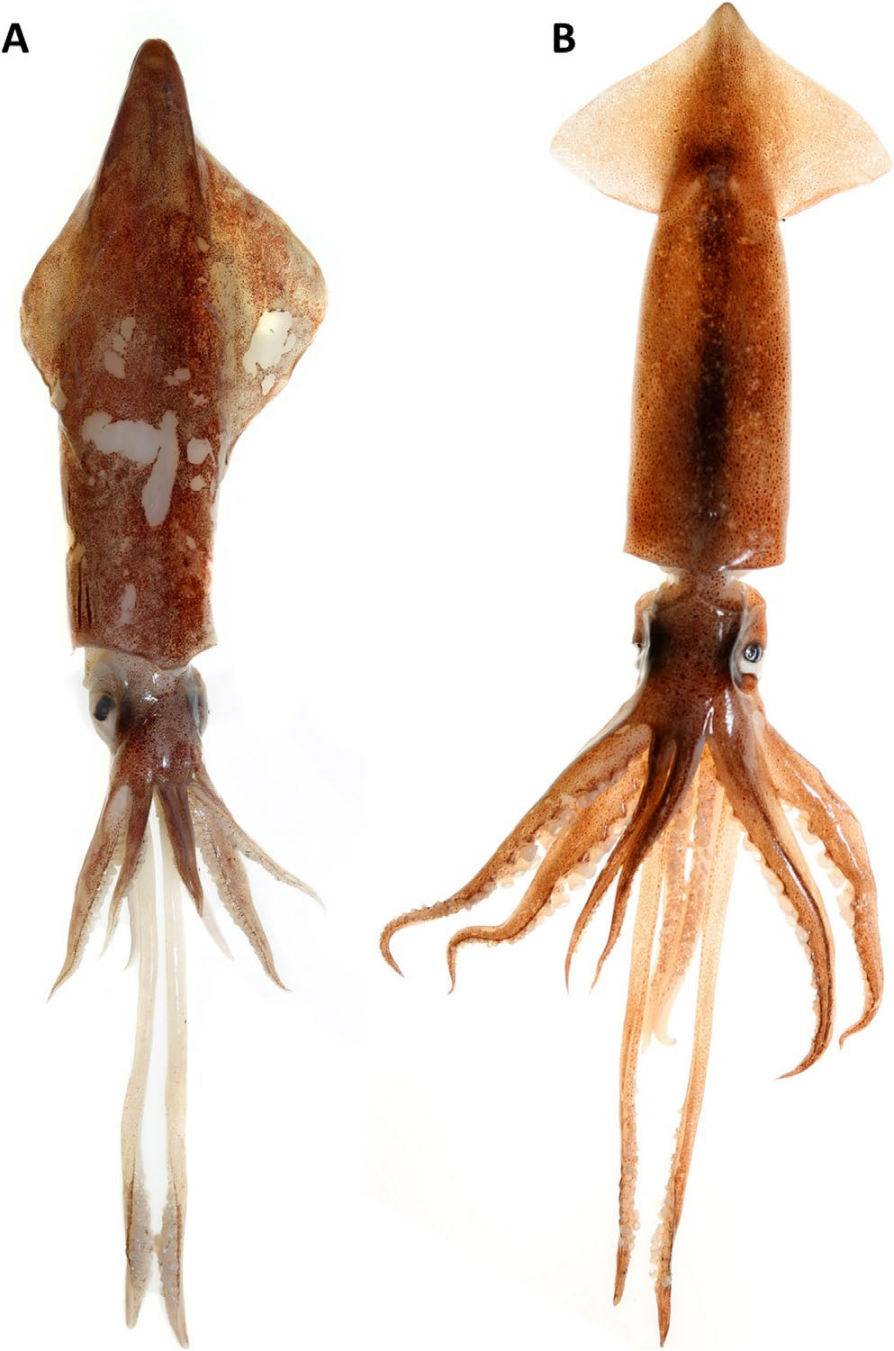
(A) Veined squid *Loligo forbesii* (Cephalopoda: Loliginidae), dorsal mantle length (DML) ca. 24 cm; (B) broadtail shortfin squid *Illex coindetii* (Cephalopoda: Ommastrephidae), DML ca. 16 cm. Thünen‐Institute/A. Schütz.

The medium‐sized veined squid *Loligo forbesii* (Loliginidae; Figure 1A) is the dominant squid species in the North Sea in terms of biomass (Jereb and Roper 2010; van der Kooij et al. 2016; Oesterwind et al. 2022). It occupies a wide range of trophic levels throughout its life cycle (Otogo et al. 2015). It is both prey of and predator on commercially important taxa, such as fish, crustaceans, and other cephalopods (Collins and Pierce 1996; Daly et al. 2001; Oesterwind et al. 2022). *L. forbesii* is also a target of commercial fisheries (Schäfer et al. 2024). The broadtail shortfin squid *Illex coindetii* (Ommastrephidae; Figure 1B) is a medium‐sized, warm‐temperate species. It has established a successful spawning stock in the North Sea since the late 2010s (Jereb and Roper 2010, Oesterwind et al. 2020, Barrett et al. 2022, Oesterwind et al. 2022, Schäfer et al. 2024). Similar to *L. forbesii* , previous studies suggest that *I. coindetii* is a generalist predator with a wide prey spectrum, both on an individual and species level (Dawe 1988; Gong et al. 2020; Oesterwind and Piatkowski 2023).

The metabolism of cephalopods is mainly protein‐based, and, consequently, the lipid and fatty acid (FA) content is relatively low in most tissues (Phillips et al. 2001). Cephalopods have a limited ability to digest, metabolize, and store lipids (Semmens 1998). Still, certain polyunsaturated FAs are essential for growth (Stowasser et al. 2006; Atayeter and Ercoşkun 2011). Exceptional in the otherwise low‐fat tissues of cephalopods is the digestive gland (DG) (Castro et al. 1992; Semmens 1998; Rosa, Costa, et al. 2005). Although the role of the DG in lipid metabolism is not fully understood, it is involved in biochemical food processing and energy storage (O'Dor and Webber 1986; Phillips et al. 2001, 2002; Swift et al. 2005; Omura and Endo 2016). The DG is the only organ in squids that contains significant amounts of nonstructural lipids and thus FAs and appears to be the preferred target organ for FA analysis (e.g., Phillips et al. 2002; Saito et al. 2014). The FAs derive from the ingested food (Castro et al. 1992, Semmens 1998, Rosa, Costa, et al. 2005) and, thus, provide information about the recent trophic history of about 2 weeks. The mantle tissue, in contrast, is a structural organ with a slow FA turnover rate (Chen et al. 2020), which may indicate long‐term or seasonal trophic habits of up to months. So far, only the FAs in the mantle tissues of North Sea *I. coindetii* and *L. forbesii* have been studied (Schäfer et al. 2024).

Animals have only a limited ability to modify FAs via bioconversion and elongation. The capabilities of trophic upgrading determine the consumer's demand for essential FAs (Monroig et al. 2013; Parrish 2013; De Carvalho and Caramujo 2018). Many FAs, for example long‐chain polyunsaturated FAs, can only be synthesized by primary producers such as phytoplankton and thus can only be obtained by a predator through its prey (Iverson 2009). Due to limited chemical modifications of FAs from ingestion to assimilation and deposition, FAs can provide information about ingested food over longer time scales of up to several weeks (Hagen 2000; Stowasser et al. 2006). This process is the basis of the fatty acid trophic marker (FATM) concept (Dalsgaard et al. 2003). FATMs are a useful tool to identify certain groups of prey organisms in a diet and to determine intraspecific diet variations (Phillips et al. 2001; Stowasser et al. 2006). However, the utility of FATM is limited as they cannot be used as taxonomic indicators at the species level (De Carvalho and Caramujo 2018). Additionally, it is also not possible to determine whether an FA originated directly from a primary producer or from its consumers (Budge et al. 2006). Therefore, FA analyses of prey spectra are most useful in addition to other parameters such as stable isotopes or visual identification of the stomach content (Phillips et al. 2001; Gong et al. 2020; Schäfer et al. 2024).

The rationale of this study is to investigate the trophic annidation of two competing squid species in the warming North Sea. The progressive overlap in the distribution range of *I. coindetii* and *L. forbesii* raises the question of whether both species compete for food in their shared North Sea habitat. This question was addressed using two complementary approaches: the biochemical analysis of the lipid content and FA composition and the visual examination of the stomach content of the squids. The biochemical approach comprised the comparison of two different organs of squid, the mantle and the DG. We test the hypothesis that both species, *I. coindetii* and *L. forbesii* , are generalist feeders, which will be reflected in significant overlaps in the FATM and the stomach contents. The results are discussed in view of the anticipated progressing food competition between both species.

## 2 Materials and Methods

### 2.1 Origin of Samples

**FIGURE 2:**
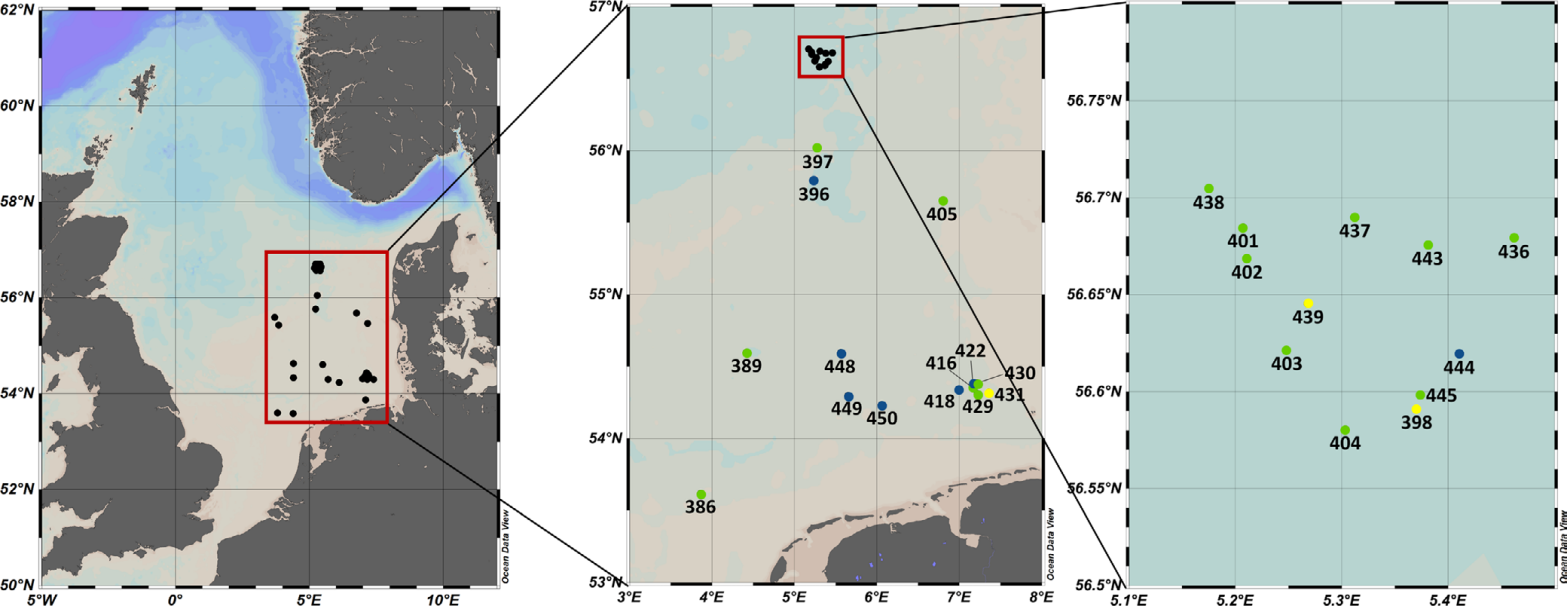
Sampling stations with station numbers of squids from this study during the WH468 North Sea cruise in summer 2023. Green: Only *Illex coindetii* from this study; blue: Only *Loligo forbesii* from this study; yellow: Both *I. coindetii* and *L. forbesii* from this study.

The squid were sampled from July 19 until August 1, 2023, in the North Sea during the WH468 cruise of the research vessel Walther Herwig III, which was part of the International Bottom Trawl Survey (IBTS) and the German Small‐scale Bottom Trawl Survey (Figure 2, Table A1). The sampling device was an otter board trawl of the type Grande Ouverture Verticale. Net geometry was monitored for the individual hauls according to the IBTS manual (ICES 2020). The hauls lasted 30 min. The squid were immediately separated from the rest of the catch and kept on ice for species identification after Oesterwind et al. (2017) and initial length measurements (measured to the lower 0.5 cm). The separated squid were frozen at −30°C and stored after the cruise at the same temperature until further processing. All procedures were conducted in accordance with European Directive 2010/63/EU on the protection of animals used for scientific purposes.

### 2.2 Sample Preparation

Two pieces of about 0.5 g of frozen mantle tissue were dissected per squid for FA analysis. The DGs were dissected from frozen squid, which were thawed as little as needed to allow incision with a scalpel, and then kept frozen until the dissection of the stomachs was performed. All samples intended for FA analysis were stored at −30°C until further processing. Like the DGs, the stomachs were dissected from the still frozen but slowly thawing squid. They were immediately transferred into reaction vials, filled with 96% ethanol (EtOH), and stored at 5°C. To ensure that the stomach content was preserved as well, incisions were made in the stomach walls prior to the transfer to the reaction vials. After the squid had thawed completely, their dorsal mantle length (DML) and wet mass were measured. Sex and maturity stage of each squid were determined following the guidelines of ICES (2010).

### 2.3 Lipid and Fatty Acid Analysis

Mantle tissue and DG samples of *Loligo forbesii* and *Illex coindetii* were examined for their lipid content and FA composition. Mantle tissue samples weighed 0.54 ± 0.21 g, and samples of the DG 0.21 ± 0.18 g. Lipid extraction followed a two‐step FA transmethylation to enable gas chromatographic (GC) separation. All chemicals were of at least HPLC‐grade.

The samples were freeze‐dried overnight in a CHRIST Alpha 1–2 LD plus freeze dryer at −20°C and 1 mbar. Sample dry mass was determined gravimetrically. Mantle tissue samples were coarsely ground. This step was not necessary for DG samples due to their paste‐like consistency. The total lipid extraction was carried out after Smedes (1999) as modified by Schäfer et al. (2024). The lipid content was expressed as a percent of the sample dry mass. The transesterification of the FAs was conducted after Schäfer et al. (2024). The FA methyl esters (FAMEs) were analyzed with an Agilent 8890 GC system equipped with a flame ionization detector (FID) and a DB Fatwax UI column (30 m × 0.25 mm × 0.25 μm). A Vici DBS PG plus hydrogen generator (Schmidlin) provided a H_2_ flow of 40 mL min^−1^ and Helium 6.0 was used as carrier gas (1.2 mL min^−1^). A split injection system with a split ratio of 1:50 and an injection volume of 1 μL was used to load the sample. The oven program was set to 180°C for 2 min, heating to 210°C with a rate of 2°C per min, and then constantly 210°C for 35 min. The FID temperature was 280°C. Retention times of the FAMEs were compared with a Supelco 37‐component FAME mixture (Sigma CRM47885), PUFA No. 1 marine source (neat, Sigma 47033) and PUFA No 3 from menhaden oil (Sigma 47085). Quality control was conducted with standard reference material for fish oil (omega‐3 and omega‐6 FAs in fish oil, Sigma NIST3275). After GC analysis, the remaining samples were stored at −20°C. Only FAs with a mean value above the respective limit of quantification after Schäfer et al. (2024) were considered as certainly determined and used for statistical analyses. The limit of quantification was specific for each FA. The content of each FA was expressed as a percent of total FAs per sample.

### 2.4 Visual Stomach Content Analysis

Visual analyses of the stomach contents were done with the help of a Zeiss Stemi 508 stereo microscope. As the storage in EtOH caused a yellowish coloration of the samples, the samples were filtered through a nylon gauze with 53 μm mesh width and rinsed with distilled water to facilitate the optical analysis. The prey composition was determined based on identifiable structures within each stomach. Several identification keys were utilized to identify different types of prey remains in the examined stomachs: Clarke (1986) for cephalopod beaks, Campana (2014) and Härkönen (1986) for sagittal otoliths, Watt et al. (1997) for fish vertebrae and premaxillae, and Hayward and Ryland (1995) for complete organisms, respectively. Each prey specimen was identified to the lowest taxon possible. After identification, the hard structures were stored separately from the remaining stomach in new 96% EtOH. Differing progress of digestion and disintegration of the stomach content hampered, in some cases, the ultimate identification of the items through visual inspection with the dissecting microscope. Some taxa could only be identified to the phylum level, whereas others could be identified to species level.

### 2.5 Sample Selection for Statistical Analysis

During the WH468 cruise, 43 *I. coindetii* and about 4000 *L. forbesii* were caught. Of these individuals, only those with DML > 8.5 cm were selected for preliminary measurements. Small individuals were excluded to focus on larger individuals with a more piscivore behavior to avoid ontogenetic variation. This size criterion was fulfilled by 42 *I. coindetii* and 30 *L. forbesii* . Of the 72 individuals, a total of 41 individuals (27 *I. coindetii* and 14 *L. forbesii* ) fulfilled the further criteria of a total FA content of ≥ 1.9 mg mL^−1^ of extract in the mantle, a nonempty stomach, and an intact gladius to allow precise DML determination. These 41 individuals (Table A2) were the subject of the present study.

### 2.6 Statistical Analysis

Microsoft Excel 2022 and RStudio version 2024.04.0 + 735 (Posit Team 2024) with R version 4.4.0 (R Core Team 2024) were used for statistical data analysis. If not stated differently, base R functions were used. The R script is provided in supplementary file S1 and the used data in supplementary files [Link], [Link], [Link], [Link], [Link], [Link]. The initial data exploration followed the protocol of Zuur et al. (2010). Data, including size and weight, lipid content and FA proportions, and stomach contents, were tested for normal distribution with the base R function Shapiro–Wilk test and for homogeneity with Levene's test using the car package (Fox and Weisberg 2019). The size and weight data were not normally distributed and not homogeneous and thus analyzed with the Wilcoxon rank sum test.

As lipid data and FA data were not normally distributed and not homogenous, generalized linear models (GLMs) with Gamma error distribution and log link function were used for analysis. Estimated marginal means (EMMs) were calculated with the emmeans package (Lenth et al. 2025). Exclusively FAs of the DG were examined in further analyses due to their higher relevance for dietary projections. Only FAs that provided at least 3% of the total FA of at least one specimen were considered. To reduce the number of NAs, one individual of *L. forbesii* and the FA C16:2 (n4) were omitted from the subsample. This resulted in nine FAs being considered for the analyses. Homogeneity of multivariate dispersion was tested. The data were square root transformed. Nonmetric multidimensional scaling (NMDS) with Bray–Curtis dissimilarity was conducted followed by an analysis of variance (ANOVA) to test for nonsignificant differences in the dispersion of the data between species. If the differences were not significant, a permutational analysis of variance (PERMANOVA) was conducted. The NMDS, ANOVA, and PERMANOVA were performed using the R packages vegan (Oksanen et al. 2024) and pairwiseAdonis (Martinez Arbizu 2020).

To compare specific prey items in the stomach contents of *I. coindetii* and *L. forbesii* , GLMs with Tweedie distribution (variance power = 1.5) and log link function using the statmod package (Smyth et al. 2023) and EMMs were used for analysis as data were not normally distributed. After testing for homogeneity of multivariate dispersion, the stomach content data were square root transformed and the steps of the NMDS and the PERMANOVAs were conducted as described for the FA data.

Data visualization was done with packages colorspace (Zeileis et al. 2009, 2020), ggplot2 (Wickham 2016), ggpubr (Kassambara 2023), and scales (Wickham, Pedersen, and Seidel 2023). Further used packages were dplyr (Wickham, François, et al. 2023), devtools (Wickham et al. 2022), tidyverse (Wickham et al. 2019), and writexl (Ooms 2023). Other softwares utilized for figure creation were GIMP version 2.10.32, and Ocean Data View version 5.6.3 (Schlitzer 2022).

Unless otherwise stated, results are presented as mean ± SD. In the portrayed box plots, the box shows the median and the interquartile range (IQR) between the 25% and 75% quartiles. The whiskers indicate data points within 1.5‐fold IQR. Individual points represent data beyond the IQR.

To identify the extent of overlap *O* of the prey spectra of *I. coindetii* and *L. forbesii* , Pianka's index of niche overlap was calculated after Pianka (1973):

$$O_{\mathrm{jk}}=\frac{\sum_{i}^{n}p_{\mathrm{ij}}\;p_{\mathrm{ik}}}{\sqrt{\sum_{i}^{n}p_{\mathrm{ij}}^{2}\sum_{i}^{n}p_{\mathrm{ik}}^{2}}}$$

where *j* refers to species *j*, *k* refers to species *k*, *p*_*ij*_ refers to the occurrence of a prey item in species *j*, and *p*_*ik*_ refers to the occurrence of a prey item in species *k*. Possible values range from zero to one. The closer the calculated value is to one, the higher the overlap in prey spectra between the examined species, with an index of one indicating a total overlap.

Levins' niche breadth *B* was calculated after Levins (1968) and Hurlbert (1978) to estimate the level of specialization of *I. coindetii* and *L. forbesii* :

$$B=\frac{1}{n\sum p_{\mathrm{xi}}^{2}}$$

where *p*_*xi*_ refers to the occurrence of a prey item in a species. If B equals the amount of prey sources, the examined species utilizes all of its resources equally. *Vice versa*, if B equals one, the examined species prefers only one type of food resource.

To calculate the trophic levels of *I. coindetii* and *L. forbesii* , the trophic levels of all identified prey items were obtained from Jiming (1982), Pauly and Christensen (1995) and FishBase (Froese and Pauly 2000; Table 1). The calculation was conducted after Gascuel et al. (2011):

$$T_{i}=1+\sum_{j}D_{\mathrm{ij}}T_{j}$$

where *T*_*i*_ refers to the trophic level of the consumer *i*, *D*_*ij*_ to the proportion of prey *j* in the diet of consumer *i*, and *T*_*j*_ is the trophic level of prey *j*. Nematoda were assumed to be parasites and not prey and were thus excluded from stomach content related calculations.

**TABLE 1:** Trophic levels of different prey items of *Illex coindetii* and *Loligo forbesii* , as reported in the references given.

Taxon	Trophic level	References
*Ammodytes*	3.3	Jiming (1982)
Ammodytidae	3.3	Jiming (1982)
Calanoida	2.2	Jiming (1982)
Cephalopoda	3.2	Pauly and Christensen (1995)
Chaetognatha	3.5	Jiming (1982)
*Clupea harengus*	3.4	FishBase (Froese and Pauly 2000)
Clupeidae	3.2	Pauly and Christensen (1995)
Crustacea	2.6	Pauly and Christensen (1995)
Cyclopoida	2.1	Jiming (1982)
Decapoda	2.6	Pauly and Christensen (1995)
Gadidae	3.8	Pauly and Christensen (1995)
Gastropoda	2.1	Pauly and Christensen (1995)
Gobiidae	2.8	Pauly and Christensen (1995)
*Gymnocephalus cernua*	3.3	FishBase (Froese and Pauly 2000)
Isopoda	2.4	Pauly and Christensen (1995)
Loliginidae	3.2	Pauly and Christensen (1995)
Paguridae	2.6	Jiming (1982)
*Trisopterus*	3.9	Jiming (1982)
Teleostei	2.8	Pauly and Christensen (1995)

## 3 Results

**FIGURE 3:**
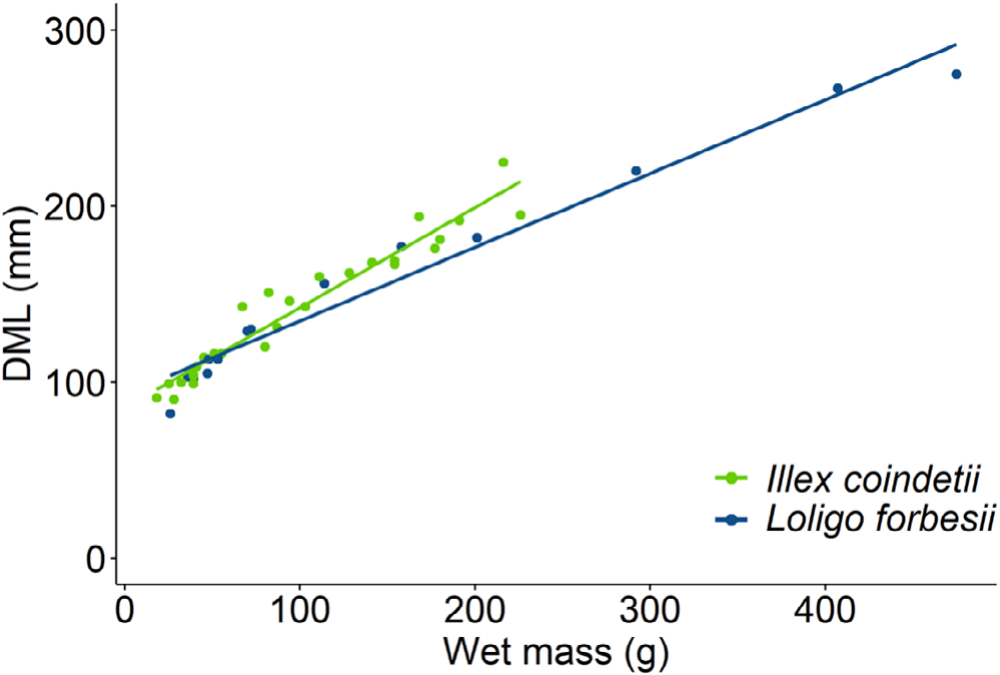
Relation between wet mass (g) and dorsal mantle length (DML, mm) of *Illex coindetii* (*n* = 27) and *L. forbesii* (*n* = 14).

The average DML and wet mass of the examined *I. coindetii* were 143 ± 37 mm and 101 ± 64 g and of the examined *L. forbesii* were 154 ± 62 mm and 146 ± 147 g. The correlation between wet mass and DML is presented in Figure 3. There was no significant difference in DML and wet mass between species (Wilcoxon rank sum test, both *p* > 0.05).

### 3.1 Total Lipids

**FIGURE 4:**
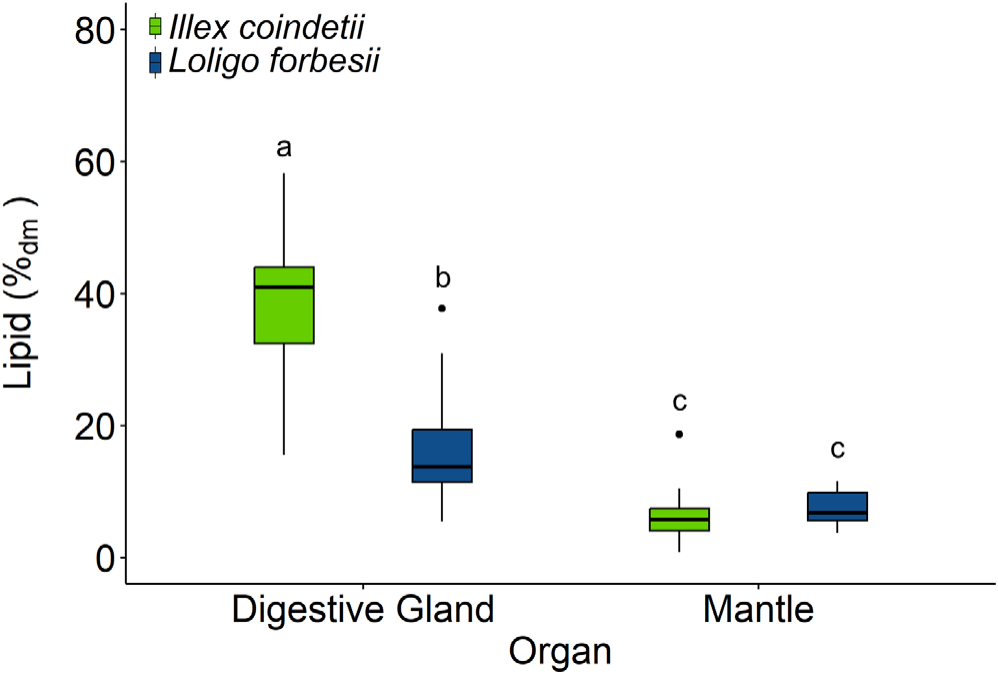
Total lipid contents in the mantle tissue and digestive glands of *I. coindetii* (*n* per organ = 27) and *L. forbesii* digestive gland (*n* = 13) and mantle tissue (*n* = 14). Different letters above the boxes indicate significant differences between data sets (generalized linear models with estimated marginal means, *p* < 0.05).

The total lipid content in the mantle of *I. coindetii* accounted on average for 6.4%_dm_ ± 3.4%_dm_, and that of *L. forbesii* for 7.4%_dm_ ± 2.6%_dm_ (Figure 4). There was no significant difference between the lipid content of the mantle of both species (GLMs with EMMs, *p* = 0.66). The average lipid content in the DG of *I. coindetii* was 38.8% ± 10.3% of the sample dry mass (%_dm_) and thus more than twice as high as that of the DG of *L. forbesii* with 16.7%_dm_ ± 9.3%_dm_ (GLM with EMMs, *p* < 0.001). Moreover, the lipid content was significantly higher in the DG than in the mantle tissue in both *I. coindetii* and *L. forbesii* (GLM with EMMs, all *p* < 0.001).

### 3.2 Fatty Acids

The FA classes saturated FAs, mono‐unsaturated FAs (MUFA), and polyunsaturated FAs (PUFA), and showed different proportions dependent on species and organ (Table 2). The share of PUFAs was always significantly higher than the share of MUFAs (GLMs with EMMs, all *p* < 0.05). The patterns of the FA classes were more similar in the DGs of both species than in the mantle tissues.

**TABLE 2:** Fatty acid contents of *Illex coindetii* and *Loligo forbesii* as a percentage of total fatty acids.

Fatty acids	*Illex coindetii*		*Loligo forbesii*	
	Digestive gland	Mantle	Digestive gland	Mantle
C14:0	3.4 ± 1.0	1.6 ± 0.3	3.3 ± 1.7	2.7 ± 0.4
C15:0	0.5 ± 0.1	—	0.6 ± 0.1	0.6 ± 0.1
C16:0	16.4 ± 2.3	26.2 ± 1.8	19.6 ± 3.5	27.1 ± 0.8
C16:1 (n7)	3.3 ± 1.1	0.7 ± 0.1	2.3 ± 1.9	0.8 ± 0.1
C16:2 (n4)	2.3 ± 1.1	—	1.1 ± 0.6	—
C17:0	0.5 ± 0.1	0.7 ± 0.1	0.8 ± 0.2	0.6 ± 0.1
C16:3 (n4)	0.4 ± 0.1	—	0.3 ± 0.1	—
C16:4 (n1)	0.1 ± 0.0	—	0.4 ± 0.4	—
C18:0	5.2 ± 1.9	6.1 ± 1.5	7.6 ± 1.7	4.8 ± 0.6
C18:1 (n9)	14.3 ± 4.4	2.7 ± 0.6	6.4 ± 4.2	3.3 ± 0.3
C18:1 (n7)	2.4 ± 0.5	1.7 ± 0.2	2.2 ± 0.5	1.9 ± 0.1
cis C18:2 (n6)	1.3 ± 0.4	—	0.7 ± 0.4	—
C18:3 (n6)	0.1 ± 0.0	—	—	—
C18:3 (n4)	0.2 ± 0.1	—	—	—
C18:3 (n3)	0.9 ± 0.3	—	0.7 ± 0.3	—
C18:4 (n3)	1.2 ± 0.5	—	0.9 ± 0.6	—
C20:0	0.3 ± 0.2	—	—	—
C20:1 (n9)	3.4 ± 2.1	3.9 ± 1.0	2.8 ± 1.2	2.5 ± 0.5
C20:2 (n6)	0.6 ± 0.2	—	0.4 ± 0.1	—
C20:3 (n6)	0.1 ± 0.0	—	—	—
C20:4 (n6)	1.0 ± 0.2	1.6 ± 0.5	1.7 ± 0.3	1.4 ± 0.6
C20:3 (n3)	0.3 ± 0.1	—	0.3 ± 0.1	—
C20:4 (n3)	0.9 ± 0.2	—	0.5 ± 0.2	—
C20:5 (n3)	9.2 ± 1.1	16.2 ± 1.5	19.7 ± 3.6	17.0 ± 0.7
C22:1 (n11)	3.3 ± 2.8	—	2.0 ± 1.7	—
C22:1 (n9)	0.4 ± 0.1	—	0.2 ± 0.1	—
C22:5 (n3)	1.4 ± 0.7	—	1.2 ± 0.4	0.7 ± 0.2
C22:6 (n3)	26.8 ± 6.6	41.2 ± 3.1	29.9 ± 4.8	37.5 ± 1.0
C24:1 (n9)	0.8 ± 0.2	—	0.5 ± 0.1	—
SFA	25.7 ± 3.3	33.2 ± 2.4	30.8 ± 3.7	35.0 ± 1.2
MUFA	27.0 ± 6.5	7.3 ± 1.6	13.5 ± 7.5	8.4 ± 0.9
PUFA	46.1 ± 6.9	58.9 ± 2.8	54.9 ± 5.7	56.0 ± 1.0

*Note:* Means ± SD. *I. coindetii* : *n* per organ = 27, *L. forbesii* : *n* per organ = 14.

The composition of FAs differed between species and organs (Table 2). The mantle tissue of *I. coindetii* yielded 10 FAs while 13 FAs were successfully determined in samples of *L. forbesii* . In both species, the most common FAs were docosahexaenoic acid (DHA, C22:6 (n3)), palmitic acid (C16:0), and eicosatetraenoic acid (EPA, C20:5 (n3)). Stearic acid (C18:0) was the only FA in the mantle that differed significantly between species (GLMS with EMMs, *p* = 0.03).

**FIGURE 5:**
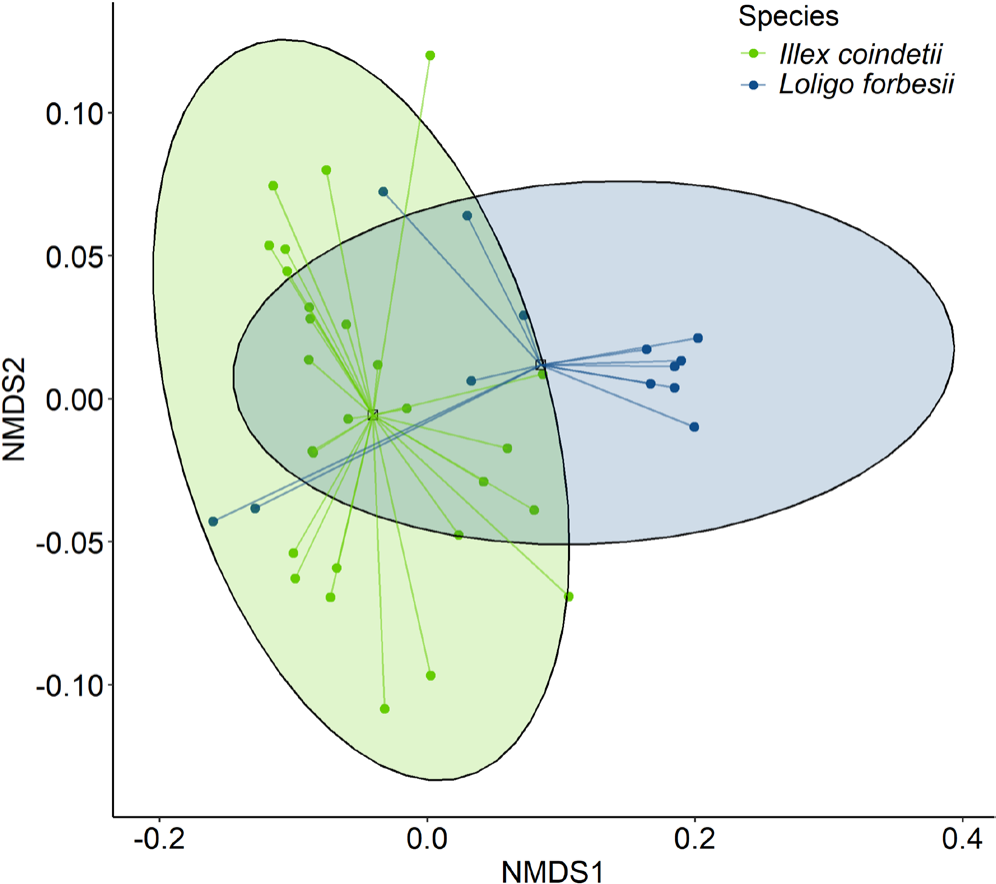
Nonmetric multidimensional scaling plot with Bray–Curtis dissimilarity of nine digestive gland fatty acids of *Illex coindetii* (*n* = 27) and *Loligo forbesii* (*n* = 13).

DG samples always contained more FAs than mantle samples. The DG of *I. coindetii* contained 29 FAs and that of *L. forbesii* 25 FAs. C22:6 (n3), C20:5 (n3), and C16:0 dominated in the DG of *L. forbesii* . DHA and C16:0 were among the most common FAs in the DG of *I. coindetii* . Of the nine DG FAs that were considered for further analyses, C16:0, C18:0, oleic acid (C18:1 (n9)), and C20:5 (n3) differed significantly between species (GLMs with EMMs, all *p* < 0.001). The NMDS plot (Figure 5) visualizes the differences between FA profiles of the DG of both species. NMDS followed by permutational analyses of variance (PERMANOVAs) showed a significant difference in the FA compositions of the DGs of *I. coindetii* and *L. forbesii* (Figure 5, *p* < 0.001, *R* ^2^ = 0.356).

### 3.3 Stomach Content

**FIGURE 6:**
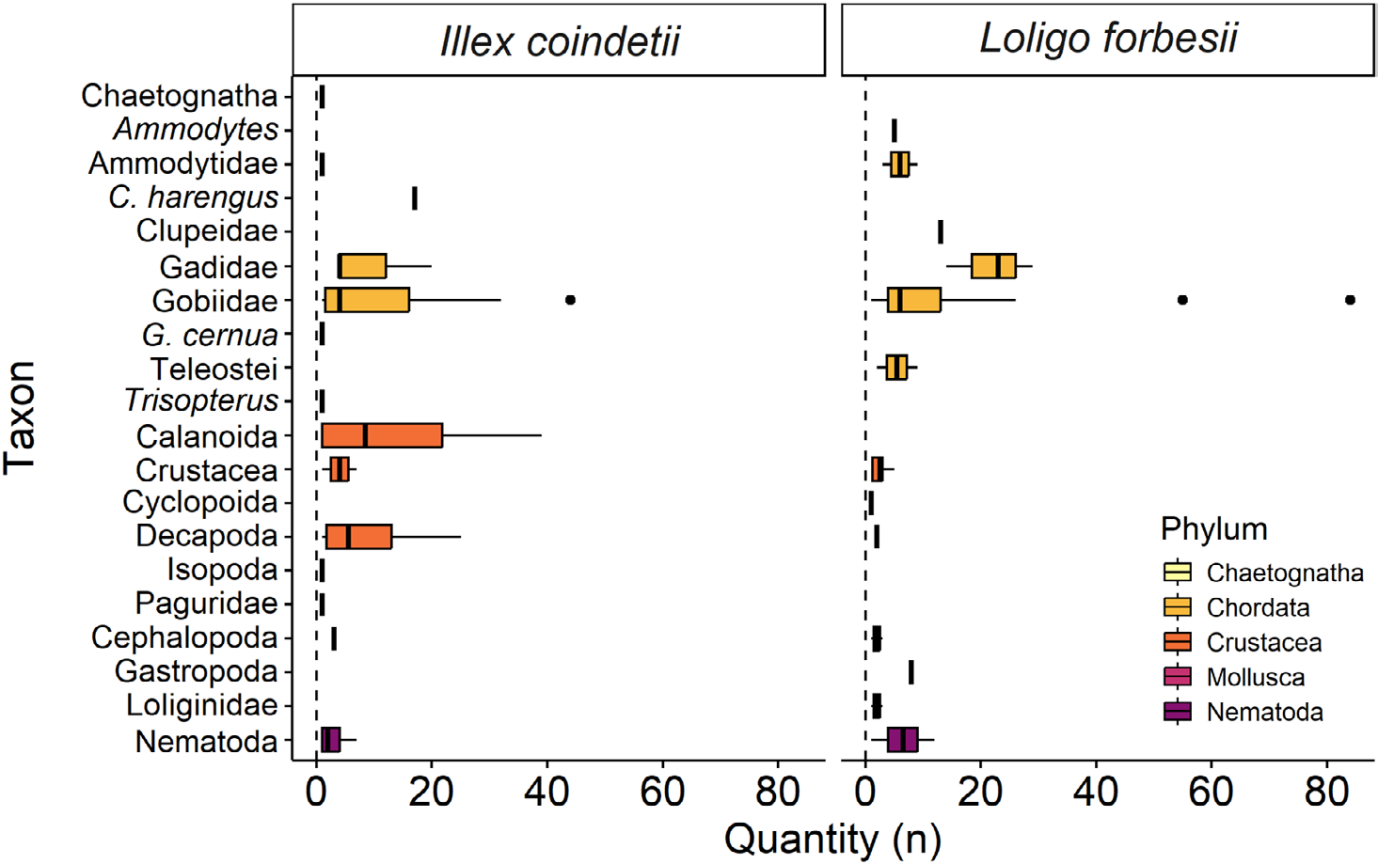
Stomach content of *Illex coindetii* (*n* = 27) and *Loligo forbesii* (*n* = 14). Quantity (*n*) in the respective squid species of items that could be related to different taxa. *C. harengus* , *Clupea harengus* , *G. cernua* , *Gymnocephalus cernua*.

The stomachs of all 41 squids, 27 individuals of *I. coindetii* and 14 *L. forbesii* , were examined for their content. Though not empty, five *I. coindetii* and four *L. forbesii* stomachs contained no identifiable structures. In the remaining stomachs, a total of 20 different taxa from five different phyla could be identified (Figure 6). Fourteen of these taxa occurred in the stomachs of *I. coindetii* and 13 in *L. forbesii* .

Teleost fishes (Chordata) represented the dominant taxon in the stomachs of *I. coindetii* and *L. forbesii* in terms of overall quantity of identified structures (items summed up in all samples, e.g., otoliths, vertebrae). The most dominant families were Gobiidae and Gadidae. In terms of the quantity of structures, there were no significant differences between Gobiidae and Gadidae in the stomachs of *I. coindetii* and *L. forbesii* (GLM with EMMs, both *p* > 0.05).

Crustacea were the second most common phylum in both squid species. Calanoida and Decapoda were the most common crustaceans in *I. coindetii* . Due to their poor condition, only very few crustacean structures could be identified to a lower taxonomic level in the stomachs of *L. forbesii* , with no clear dominance of any lower crustacean taxon.

The third most frequent phylum in *I. coindetii* was Nematoda, which was considered as parasites and could not be further identified. In *L. forbesii* , the third most frequent phylum was mollusks. Different from the findings in *I. coindetii* , the mollusks in the stomachs of *L. forbesii* did not completely consist of cephalopods but also, to a smaller degree, of gastropods. Chaetognatha only occurred in a single stomach of *I. coindetii* and was completely absent in individuals of *L. forbesii* . There was no significant difference in the overall prey composition between the two species (PERMANOVA, *p* = 0.415, *R* ^2^ = 0.032).

### 3.4 Trophic Indices

Pianka's index of niche overlap of *I. coindetii* and *L. forbesii* was determined using the proportions of prey items in the overall prey composition. It was calculated with each identified prey taxon regardless of taxonomic level and only with the phylum level of prey items, resulting in values of 0.93 and 0.97, respectively. These values indicate a very similar trophic niche for the two squid species.

Levins' niche breadth was calculated with the same data as Pianka's index: the proportions of prey items in the overall prey on the phylum (*n* = 5) level and on the most detailed (*n* = 20) level. This resulted in an index of 1.87 on the phylum level and 2.82 on the most detailed level for *I. coindetii* . For *L. forbesii* , the results were 1.22 for the phylum level and 2.45 for the most detailed level, indicating a less even use of different prey taxa in its diet compared to *I. coindetii* . However, this index did not provide information on whether *I. coindetii* and *L. forbesii* fed on the same prey taxa.

The trophic levels of *I. coindetii* and *L. forbesii* were calculated from the proportions of each prey taxon in their diet based on numerical abundance and the trophic levels of the prey taxa as obtained from the literature. *I. coindetii* had a calculated trophic level of 3.80 and *L. forbesii* had a calculated trophic level of 4.00, implying a mesopredatory role for both species.

## 4 Discussion

### 4.1 Lipid Content and Fatty Acid Composition

The principal component analysis (PCA) of the FAs of the mantle showed a less clear group separation between the two species than previously reported by Schäfer et al. (2024). This may be due to the sampling season. Schäfer et al. (2024) examined a winter cohort while we investigated a summer cohort in the present study. Thus, the specimens of both studies may have been exposed to different prey spectra (Oesterwind and Piatkowski 2023).

Both species, *I. coindetii* and *L. forbesii* , showed considerably higher lipid contents in the DG than in the mantle tissue. The exact function of the DG of squid is not clear, but it appears to be an important energy storage organ involved in the regulation of lipid metabolism (Castro et al. 1992; Phillips et al. 2001, 2002; Rosa, Costa, et al. 2005).

In addition to the higher lipid content, the DGs of either species also displayed a higher variety of FAs than the mantle tissue, both in terms of number and composition of FAs. Moreover, the FA composition of the DG differed more between species than the FA compositions of the mantle tissues. Apparently, the FA profile of the DG is more strongly influenced by the FA composition of the consumed prey than the mantle tissue (Semmens 1998; Phillips et al. 2001, 2002). The mantle, on the other hand, forms a structural element with a well‐defined set of phospholipids (Phillips et al. 2001). Even within the same species, the FA composition of the DG may vary depending on the diet of the individuals (Stowasser et al. 2006; Gong et al. 2020). A high degree of individual dietary specialization has been identified in some squid species, for example, *Illex argentinus* , especially in mature, reproducing individuals (Lin et al. 2025). As the majority of the examined *I. coindetii* were mature, their individual level of dietary specialization may have been high, influencing their FA compositions. This individual variance may decrease the utility of some FATM on the species level. Overall, polyunsaturated FAs (PUFAs) dominated the FAs in both organs of *I. coindetii* and *L. forbesii* . Docosahexaenoic acid, C22:6 (n3), was the major FA, which is in line with previous studies (e.g., Phillips et al. 2001; Gong et al. 2020; Schäfer et al. 2024).

#### 4.1.1 Fatty Acid Trophic Markers

Some of the DG FAs identified in our study can be used as FATMs. The content of stearic acid C18:0 was significantly higher in the DG of *L. forbesii* than in *I. coindetii* with 7.6% ± 1.7% vs. 5.2% ± 1.9% of total FAs. Feeding experiments with the Atlantic brief squid *Lolliguncula brevis* (Loliginidae) showed that this FA is more abundant in individuals that fed on fish compared to individuals with a crustacean‐based diet (Stowasser et al. 2006). Thus, this significant difference indicates that our analyzed *L. forbesii* had a more fish‐based diet than *I. coindetii* .

Oleic acid C18:1 (n9) was significantly more abundant in *I. coindetii* than in *L. forbesii* (14.3 ± 4.4 vs. 6.4% ± 4.2% of total FAs). The feeding experiments with *L. brevis* by Stowasser et al. (2006) showed that this FA is more prominent in individuals with a more crustacean‐based diet, which corresponds with our findings for C18:0.

The content of gondoic acid C20:1 (n9) showed no significant difference between *I. coindetii* and *L. forbesii* . Its moderate amount in the DG of both species may indicate the presence of copepods in the diet of both species (Budge et al. 2006; Schäfer et al. 2024). As copepods are not common prey items of squid of the analyzed size, this FATM likely reflects secondary ingestion, meaning that the squid likely fed on prey that had previously fed on copepods (Ibáñez et al. 2021). Though not significant, the content of C20:1 (n9) was higher in the DG of *Illex coindetii* , which is in agreement with the higher abundance of copepods in the stomach contents of this species. Dietary specialization of individuals of this species may have prevented significant differences in the FA content between species (Lin et al. 2025).

Eicosapentaenoic acid C20:5 (n3) belongs to the most characteristic FAs in cephalopods (Sinanoglou and Miniadis‐Meimaroglou 1998; Atayeter and Ercoşkun 2011). Generally, this FA seems to be important for aquatic trophic transfer and the health of several animal taxa (Müller‐Navarra et al. 2000; Parrish 2013; Saito et al. 2014). It made up a large proportion of the FAs of both *I. coindetii* and *L. forbesii* with 9.2 ± 1.1 and 19.7% ± 3.6% of total FAs, respectively, and was significantly higher in *L. forbesii* . This FA is characteristic of invertebrates that feed on single‐celled algae, indicating the presence of invertebrate herbivores in the diet of the two squid species (Sinanoglou and Miniadis‐Meimaroglou 1998). Thus, like C20:1 (n9), this FATM may also indicate secondary ingestion. C20:5 (n3) is also quite abundant in clupeiform fishes such as herring *Clupea harengus* (Nedenskov Jensen et al. 2007), which corresponded with the occurrence of clupeid items in the stomachs of both squid species. However, C20:5 (n3) is also elevated in migratory fish and occurs in almost all species and taxa (Sinanoglou and Miniadis‐Meimaroglou 1998; Saito et al. 2005). Therefore, drawing clear conclusions from the content of this FATM is difficult.

Docosahexaenoic acid C22:6 (n3) is one of the major PUFAs in cephalopods and can be related to a diet of either crustaceans, fishes, or cephalopods (Sinanoglou and Miniadis‐Meimaroglou 1998; Stowasser et al. 2006; Kelly and Scheibling 2012). In cephalopods, C22:6 (n3) may also be synthesized from C22:5 (n3) via desaturation (Monroig et al. 2013). Thus, its use as a FATM is limited. There were no significant differences in the content of this FA between *I. coindetii* and *L. forbesii* . However, it is comparatively abundant in clupeids, e.g., *C. harengus* (Nedenskov Jensen et al. 2007), which occurred in the stomachs of both squid species.

Overall, the FA compositions of the two squid species indicate a wide variety of prey items, including fishes, crustaceans, copepods, herbivorous invertebrates, and cephalopods, though some of these may be the result of secondary ingestion (Ibáñez et al. 2021). These prey items also appeared in the stomach content analyses for both species, and their occurrence is in accordance with the reports of other studies (e.g., Sinanoglou and Miniadis‐Meimaroglou 1998; Budge et al. 2006; Stowasser et al. 2006; Kelly and Scheibling 2012; Schäfer et al. 2024). While the FA trophic markers indicate some overlaps in the prey source of both species, the proportions of several prey items appear to differ between *I. coindetii* and *L. forbesii* .

### 4.2 Stomach Contents

Teleost fishes and crustaceans dominated the diets of *I. coindetii* and *L. forbesii* in this study. This is in agreement with findings in several other squid species (e.g., Mangold 1983; Rodhouse and Nigmatullin 1996; Otogo et al. 2015; Oesterwind and Piatkowski 2023). The occurrence of mollusks, mainly cephalopods, in both species, and chaetognaths in the stomachs of *I. coindetii* matches with the stomach contents of *I. coindetii* and *L. forbesii* caught off western Spain (Rocha et al. 1994; Rasero et al. 1996). The diet of *L. forbesii* was more fish‐based compared to that of *I. coindetii* .

Squid beaks found in the stomachs of *L. forbesii* belonged to the family Loliginidae. As none of these beaks could be identified to genus or species level, it is not possible to conclude whether they result from cannibalism. Though cannibalistic behavior has been reported for *L. forbesii* , other loliginids such as *Alloteuthis* spp. and the common squid *Loligo vulgaris* are also known prey organisms of this species (Oesterwind and Piatkowski 2023).

Nematodes found in the stomachs were probably parasites and not prey (Pascual et al. 1996). They are common parasites of cephalopods and have been previously identified in both *I. coindetii* and *L. forbesii* (Hochberg 1983; Smith 1984; Pascual et al. 1996). Unexpected was the lack of polychaete remains during visual stomach content analyses as polychaetes have been detected as common prey items of *L. forbesii* in the same size ranges as the individuals examined here (Collins and Pierce 1996).

Due to their generalist nature, *I. coindetii* and *L. forbesii* seem to feed on whatever prey is available as long as they can overpower it (Rasero et al. 1996; Rosa, Costa, et al. 2005; Oesterwind et al. 2020; Ibáñez et al. 2021). This was indicated by our findings and also by other studies (e.g., Rasero et al. 1996; Oesterwind and Piatkowski 2023). The nonselective predation corresponds with the relatively wide Levins' niche breadth calculated for both squid species. Correspondingly, the PERMANOVA of prey taxa, as well as the high value for Pianka's index of niche overlap of *I. coindetii* and *L. forbesii* (0.97 at the highest and 0.93 at the lowest taxonomic resolution of the prey taxa) shows a strong overlap between the two squid species. As *I. coindetii* is still fairly new to the North Sea and only established successful breeding populations in the late 2010s (Oesterwind et al. 2022), these strong overlaps may indicate competition between the two squid species.

### 4.3 Limitations of Visual Stomach Content Analysis

Visual stomach content analysis can provide detailed insights into the prey of an individual, but, especially in cephalopods, it also entails challenges and limitations (Ibáñez et al. 2021). One of the biggest limitations of this method is that it can only give insight into the most recent meals, as digestion rates in cephalopod stomachs are quite high, often yielding empty stomachs (Oesterwind 2011; Martínez‐Baena et al. 2016; Ibáñez et al. 2021). As squid use their chitinous beaks to masticate their prey before ingestion, identifiable structures are often destroyed, leading to an underestimation of certain prey items (Rodhouse and Nigmatullin 1996). Additionally, the digestion rates of different structures differ (Santos et al. 2001). Cephalopod tissues, for example, are digested at a faster rate than fish tissues (Santos et al. 2001). Rates of erosion of hard parts are species‐specific and may cause a bias toward species with larger and more robust hard structures in visual stomach content analysis (Ibáñez et al. 2021). Furthermore, the digestion rates of squid are far from constant (Hyslop 1980). They are influenced by factors like the amount of prey and the number of hard structures in it, prey lipid content, periods of food deprivation, and water temperature (Hyslop 1980). Some hard parts like cephalopod beaks remain mostly undigested and may accumulate in the stomach before egestion, potentially overestimating the importance in a diet (Santos et al. 2001). Furthermore, cephalopods may reject the heads of fish and thereby prevent the ingestion of the otoliths (Collins and Pierce 1996). Moreover, it is not possible to identify whether, for example, two otoliths, vertebrae, or eyes belonging to the same taxon also belonged to the same ingested individual, hampering estimations of the ingested number of individuals.

During sampling, cephalopods may feed in the net on species that they usually may not prey on, obscuring the natural prey spectrum (Phillips et al. 2001). Additionally, it is not possible to differentiate secondarily ingested prey, i.e., the prey of the prey, from primarily ingested specimens via visual stomach content analysis (Ibáñez et al. 2021). These so‐called transit food items can be abundant, for example occurring in up to 43.4% of stomachs of the neon flying squid *Ommastrephes bartamii* (Ommastrephidae; Nigmatullin et al. 2009; Ibáñez et al. 2021). Transit food items may include copepods and gastropods (Nigmatullin et al. 2009). In our study, this may have been the fate of benthic gastropods in the stomachs of *L. forbesii* and of calanoids in *I. coindetii* .

### 4.4 Implications for North Sea Food Webs

The calculated trophic levels for *I. coindetii* and *L. forbesii* of 3.80 and 4.00, respectively, were slightly higher than the trophic level of 3.2, which Pauly and Christensen (1995) reported for squids in “non‐tropical shelf systems”. They were also higher than the range (2.17–3.09 for *I. coindetii* and 2.50–3.85 for *L. forbesii* ) estimated by Oesterwind (2011) for North Sea squids. Otogo et al. (2015) estimated a trophic level of 4.15 for *L. forbesii* , which more closely resembles the data presented here. However, calculated trophic levels represent more of a snapshot, whereas the natural trophic level of an individual changes during ontogenesis with the size, but also with the habitat, and the available prey spectra (Pauly and Christensen 1995; Rodhouse and Nigmatullin 1996). Overall, the squid species of the current study seems to have occupied the position of mesopredators and thus may hold a key position in the North Sea food web.

As cephalopods in general, including *I. coindetii* and *L. forbesii* , seem to benefit from current climate changes and possibly also from the depletion of finfish as their predators, they have become more abundant in the North Sea over the last century, and certain species, particularly over the last decades (Piatkowski et al. 2001; Oesterwind et al. 2022). The identified prey of *I. coindetii* and *L. forbesii* comprised commercially exploited taxa such as clupeids, gadids, and decapods (ICES 2022). Several finfish species are considered overexploited in European waters (Pierce et al. 2008; ICES 2022). With their apparent role as mesopredators, the prosperity of *I. coindetii* and *L. forbesii* in the North Sea may entail advantages and disadvantages for the local fish populations. On one hand, the two squid species feed on smaller fish, including the recruits of larger fish species (Oesterwind 2011), which could increase the pressure on already overexploited stocks. On the other hand, *I. coindetii* and *L. forbesii* may be used as a food resource by several other taxa, including fishes, seabirds, marine mammals, and other cephalopods (Clarke 1996; Croxall and Prince 1996; Klages 1996; Smale 1996; Oesterwind and Piatkowski 2023). Though not rich in lipids and thus FAs, the nutritional value of these squids may lie in their high protein content, which can reach up to 73%_dm_ in the muscle tissue of *L. vulgaris* (Rosa, Pereira, and Nunes 2005).

This nutritional value also explains the relevance of squids as a human food source. *I. coindetii* is mainly commercially exploited in the Mediterranean and the North‐eastern Atlantic, including regions close to the North Sea such as the southern Celtic Sea and the Bay of Biscay, and is caught both as by catch and in targeted fisheries (Sánchez et al. 1998; Arvanitidis et al. 2002; Ceriola et al. 2007). In the North Sea, its occurrence as by‐catch is increasing (ICES 2023). Today, there are even targeted fisheries for *L. forbesii* in the North Sea, which, together with *L. vulgaris* , now forms one of the commercially most exploited squid species in Europe (Pierce et al. 1998; Göpel et al. 2022; Laptikhovsky et al. 2022; Oesterwind et al. 2022).

While the general landings of cephalopods, including squids, have been increasing since the 1950s, there is some evidence of negative effects of this commercial exploitation of squid stocks (Chen et al. 2006; Pierce et al. 2013). *L. forbesii* stocks, for example, started to decline in Portuguese and French waters in the 2000s (Chen et al. 2006). If more fisheries take advantage of the commercial value of squid, the current trend of their overall stock increase may be limited due to rising fishing pressure.

## 5 Conclusion

Combined analysis of DG FAs and stomach contents allowed a more comprehensive overview of the prey spectrum of the examined squid species, *Illex coindetii* and *Loligo forbesii* . These complementary methods imply significant similarities and overlaps in the prey spectra of both species. Accordingly, both species are potential competitors for food. With continuous warming, *I. coindetii* may become more abundant in the North Sea, and food competition with *L. forbesii* may hence increase significantly. As a result, predation between both species may also increase and probably alter food web interactions. Thus, more detailed investigations of the trophic development and the predator–prey interaction of *I. coindetii* and *L. forbesii* throughout their life cycles will be beneficial to understand changing food web dynamics. To properly assess the interactions between these two species, including the mechanisms of their competition or coexistence, future research could highly benefit from combining biochemical analyses, such as FA and stable isotope analysis, with visual and genetic stomach content analyses. In addition to that, more intensive studies on coexistence mechanisms between *I. coindetii* and *L. forbesii* in the North Sea are required.

## Author Contributions

**Hanna Rittinghaus:** conceptualization (supporting), data curation (lead), formal analysis (lead), methodology (supporting), visualization (lead), writing – original draft (lead), writing – review and editing (equal). **Reinhard Saborowski:** conceptualization (equal), data curation (supporting), supervision (equal), writing – review and editing (equal). **Ulrike K. R. Kammann:** methodology (equal), writing – review and editing (equal). **Daniel Oesterwind:** methodology (equal), writing – review and editing (equal). **Anne F. Sell:** conceptualization (lead), formal analysis (supporting), resources (lead), supervision (equal), writing – review and editing (equal).

## Conflicts of Interest

The authors declare no conflicts of interest.

## Supporting information


**Data S1:** R Script.

**Data S2:** Proportions of prey phyla in the stomach contents of *Illex coindetii* and *Loligo forbesii*.

**Data S3:** Lipid and fatty acid data of *Illex coindetii* and *Loligo forbesii*.

**Data S4:** Amounts of prey taxa hard structures in the stomachs of *Illex coindetii* and *Loligo forbesii*.

**Data S5:** Proportions of prey taxa in the stomach contents of *Illex coindetii* and *Loligo forbesii*.

**Data S6:** Amounts of prey taxa in the stomachs of *Illex coindetii* and *Loligo forbesii*.

**Data S7:** Trophic levels of prey taxa of *Illex coindetii* and *Loligo forbesii*.

## Data Availability

The data for this study are available on Open Agrar: Rittinghaus, H., Saborowski, R., Kammann, U. K. R., Oesterwind, D., and Sell, A. F. (2025). *Fatty acid patterns and stomach contents of two North Sea squid species, Loligo forbesii and Illex coindetii* ‐ [dataset]. https://doi.org/10.3220/253‐2025‐28. The R script is provided in the Supporting Information as file S1 and the relevant data subsets are provided in Appendix [Link], [Link], [Link], [Link], [Link], [Link]. https://www.openagrar.de/receive/openagrar_mods_00106542?accesskey=stomach2025L

## Appendix A

**TABLE A1:** Station overview.

Station	Latitude [°E]	Longitude [°N]	Depth [m]	Haul duration [min]	Species	Sample ID
386	53.61617	3.8615	37.9	30	*Illex coindetii*	HR34
389	54.59733	4.41667	50.8	30	*Illex coindetii*	HR29, HR51
396	55.794	5.2335	53.6	30	*Loligo forbesii*	HR59
397	56.01833	5.285	42.2	30	*Illex coindetii*	HR64
398	56.591	5.37017	56.8	30	*Loligo forbesii*	HR07
398	56.591	5.37017	56.8	30	*Illex coindetii*	HR63
401	56.68467	5.2075	61.0	30	*Illex coindetii*	HR31, HR32
402	56.66883	5.21067	60.7	30	*Illex coindetii*	HR44, HR46
403	56.6215	5.248	60.1	30	*Illex coindetii*	HR65
404	56.5805	5.3035	58.8	30	*Illex coindetii*	HR43
405	55.65467	6.7955	36.0	30	*Illex coindetii*	HR24
416	54.36117	7.16867	40.8	30	*Illex coindetii*	HR37
418	54.34183	6.99367	39.0	30	*Loligo forbesii*	HR57
422	54.38567	7.17517	39.7	30	*Loligo forbesii*	HR03, HR04, HR10, HR16
429	54.31167	7.22017	40.1	30	*Illex coindetii*	HR22
430	54.38383	7.21683	41.9	30	*Illex coindetii*	HR41
431	54.3195	7.35667	44.6	30	*Loligo forbesii*	HR02
431	54.3195	7.35667	44.6	30	*Illex coindetii*	HR49
436	56.67917	5.46167	57.7	30	*Illex coindetii*	HR50
437	56.69	5.31167	59.5	30	*Illex coindetii*	HR35, HR62
438	56.70467	5.175	61.8	30	*Illex coindetii*	HR27, HR47, HR48, HR61, HR72
439	56.6455	5.268	60.6	25	*Loligo forbesii*	HR06
439	56.6455	5.268	60.6	25	*Illex coindetii*	HR21
443	56.67567	5.38133	58.2	28	*Illex coindetii*	HR66
444	56.61967	5.411	57.2	20	*Illex coindetii*	HR68
445	56.59817	5.37383	58.3	20	*Illex coindetii*	HR33
448	54.59683	5.56317	43.7	30	*Loligo forbesii*	HR20
449	54.298	5.65	40.5	30	*Loligo forbesii*	HR55, HR56
450	54.234	6.05667	39.1	30	*Loligo forbesii*	HR05

*Note:* Overview of different parameters of the sampled stations from cruise WH468, summer 2023.

**TABLE A2:** Sample overview. Analyzed samples from cruise WH468, summer 2023.

ID	Station	Species	DML [mm]	Wet mass [g]	Sex	Maturity stage	Stomach fullness
HR21	439	*Illex coindetii*	109	41	M	3a	2
HR22	429	*Illex coindetii*	160	111	F	3a	1
HR24	405	*Illex coindetii*	146	94	F	3a	1
HR27	438	*Illex coindetii*	225	216	F	3a	3
HR29	389	*Illex coindetii*	151	82	F	3a	1
HR31	401	*Illex coindetii*	195	226	M	3a	3
HR32	401	*Illex coindetii*	169	154	M	3a	4
HR33	445	*Illex coindetii*	162	128	M	3a	1
HR34	386	*Illex coindetii*	168	141	F	3a	4
HR35	437	*Illex coindetii*	167	154	M	3a	3
HR37	416	*Illex coindetii*	143	103	M	3a	1
HR41	430	*Illex coindetii*	131	87	M	3a	2
HR43	404	*Illex coindetii*	181	180	M	3a	2
HR44	402	*Illex coindetii*	194	168	F	3a	3
HR46	402	*Illex coindetii*	143	67	F	2b	1
HR47	438	*Illex coindetii*	176	177	F	3a	1
HR48	438	*Illex coindetii*	192	191	M	3a	1
HR49	431	*Illex coindetii*	120	80	M	3a	1
HR50	436	*Illex coindetii*	114	45	M	3a	1
HR51	389	*Illex coindetii*	116	55	M	3a	1
HR61	438	*Illex coindetii*	116	51	M	3a	1
HR62	437	*Illex coindetii*	99	39	M	3a	1
HR63	398	*Illex coindetii*	91	18	F	2a	0
HR64	397	*Illex coindetii*	99	25	F	2b	1
HR65	403	*Illex coindetii*	90	28	M	2b	1
HR66	443	*Illex coindetii*	100	32	M	3a	2
HR72	438	*Illex coindetii*	104	39	M	3b	2
HR02	431	*Loligo forbesii*	156	114	M	2a	1
HR03	422	*Loligo forbesii*	130	72	F	2a	1
HR04	422	*Loligo forbesii*	129	70	M	2a	1
HR05	450	*Loligo forbesii*	177	158	M	2a	4
HR06	439	*Loligo forbesii*	182	201	F	3a	2
HR07	398	*Loligo forbesii*	220	292	F	3a	2
HR10	422	*Loligo forbesii*	275	475	M	2a	1
HR16	422	*Loligo forbesii*	267	407	M	2b	2
HR20	448	*Loligo forbesii*	103	36	M	2a	1
HR55	449	*Loligo forbesii*	113	53	F	1	1
HR56	449	*Loligo forbesii*	102	39	M	2a	1
HR57	418	*Loligo forbesii*	105	47	M	2a	1
HR59	396	*Loligo forbesii*	113	48	M	2a	2
HR68	444	*Loligo forbesii*	82	26	M	1	0

*Note:* Stomach fullness levels: 0 = empty, 1 = 1%–25% filling degree, 2 = 25%–50%, 3 = 50%–75%, 4 = 75%–100%.

Abbreviations: DML, dorsal mantle length; F, female; M, male.
